# COVID-19 and the risk of neuromyelitis optica spectrum disorder: a Mendelian randomization study

**DOI:** 10.3389/fimmu.2023.1207514

**Published:** 2023-07-27

**Authors:** Dongren Sun, Qin Du, Rui Wang, Ziyan Shi, Hongxi Chen, Hongyu Zhou

**Affiliations:** Department of Neurology, West China Hospital, Sichuan University, Guo Xuexiang, Chengdu, China

**Keywords:** COVID-19, causal effect, Mendelian randomization, NMOSD COVID-19, NMOSD

## Abstract

**Background:**

An increasing number of studies have elucidated a close nexus between COVID-19 phenotypes and neuromyelitis optica spectrum disorder (NMOSD), yet the causality between them remains enigmatic.

**Methods:**

In this study, we conducted a Mendelian randomization (MR) analysis employing summary data sourced from genome-wide association studies (GWAS) pertaining to COVID-19 susceptibility, hospitalization, severity, and NMOSD. The primary MR analysis employed the Inverse variance weighted (IVW) approach, which was supplemented by MR-Egger, weighted median, simple mode, and weighted mode methods. We implemented various sensitivity analyses including Cochran’s Q test, MR-PRESSO method, MR-Egger intercept, leave-one-out analysis, and funnel plot.

**Results:**

The MR results demonstrated a nominal association between COVID-19 susceptibility and the risk of AQP4+ NMOSD, as evidenced by the IVW method (OR = 4.958; 95% CI: 1.322-18.585; P = 0.018). Conversely, no causal association was observed between COVID-19 susceptibility, hospitalization, or severity and the increased risk of NMOSD, AQP4-NMOSD, or AQP4+ NMOSD. The comprehensive sensitivity analyses further bolstered the robustness and consistency of the MR estimates.

**Conclusion:**

Our findings provide compelling evidence for a causal effect of COVID-19 phenotype on AQP4+ NMOSD, shedding new light on the understanding of the comorbidity between COVID-19 and NMOSD.

## Introduction

Coronavirus disease 2019 (COVID-19), caused by severe acute respiratory syndrome coronavirus 2 (SARS-CoV-2), has triggered a global panic and posed a significant public health threat (1). The phenotype of COVID-19 including susceptibility, hospitalization and severity showed a high degree of heterogeneity (2). Specifically, most patients presented as asymptomatic, and a small proportion required hospitalization or even death. Changes in the neurological and psychiatric sequelae of COVID-19 survivors were tracked (1). The COVID-19 pandemic spans less than four years to date. Therefore, the physical and mental effects of COVID-19 over a longer timeline are still unknown.

Accumulating evidence suggests that SARS-CoV-2 can penetrate the blood-brain barrier and multiple pro-inflammatory cytokines such as IL-6, IL-10, and IFN-γ, trigger a cytokine storm that induces immune dysregulation leading to CNS demyelinating lesions (3–5). These factors are closely related to neuromyelitis optica spectrum disorder (NMOSD). A growing body of case reports or series has documented the emergence of NMOSD subsequent to COVID-19 infection or COVID-19 vaccination (6–9). Although these studies show a clear chronological sequence, it remains uncertain whether COVID-19 infection caused or triggered latent NMOSD. Clarifying the causal relationship between them is urgent in the COVID-19 era.

**Figure 1:**
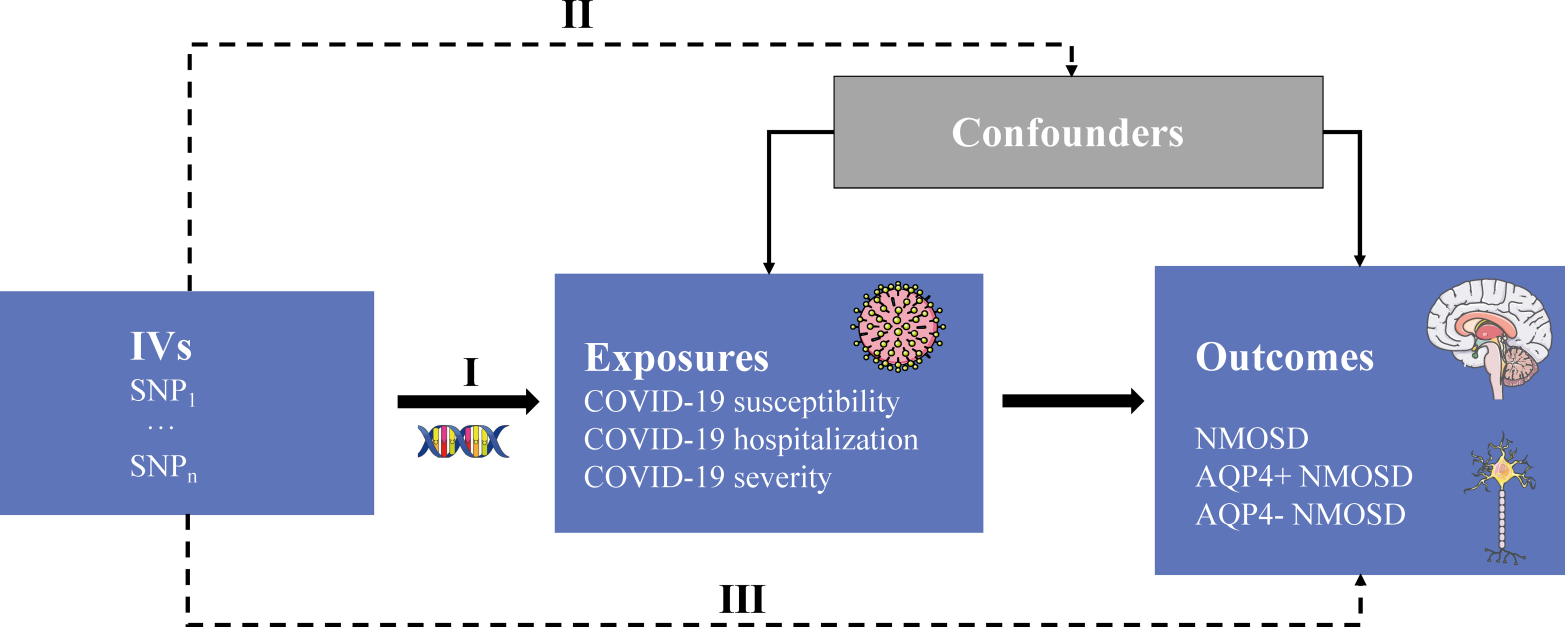
Study overview. IVs, instrumental variables; SNP, single nucleotide polymorphism; AQP4, aquaporin-4 antibody; +/-, positive/negative; NMOSD, neuromyelitis optica spectrum disease. Three core assumptions: (I) genetic variants that are IVs must be strongly associated with the exposure of interest; (II) SNPs should not be associated with any potential confounders; and (III) SNPs can only influence outcome trait through risk factors that represent the exposure not through other causal pathways.

Mendelian randomization (MR) is a robust approach employed to evaluate the causal relationship between exposure factors and outcomes, leveraging single nucleotide polymorphisms (SNPs) as instrumental variables (IVs) (10, 11). The utilization of the MR approach offers notable advantages in mitigating residual confounding, as the random allocation of genetic variants during conception ensures minimal association with confounding factors. Moreover, this method effectively mitigates the issue of reverse causality, as the genetic variations utilized to approximate the effects of exposure remain unaffected by the occurrence and progression of the outcome (12). Herein, we applied a two-sample MR approach to investigate the causal effects of COVID-19 susceptibility, hospitalization, and severity on NMOSD. The overall design flow of this study is shown in **Figure 1**.

## Methods

### Data sources and genetic instruments

#### Exposure sources

##### COVID-19

We used the most current available data pertaining to COVID-19 phenotypes, sourced from the COVID-19 Host Genetics Initiative (2) (RELEASE 7, updated April 2022). This comprehensive dataset encompasses three distinct components: COVID-19 susceptibility, COVID-19 hospitalization, and COVID-19 severity. Notably, COVID-19 susceptibility involved the analysis of 159,840 cases and 2,782,977 controls, with reliable SARS-CoV-2 infection serving as the defining criterion. The examination of COVID-19 hospitalization encompassed 44,986 cases and 2,356,386 controls, focusing on patients who required hospitalization due to COVID-19. Additionally, the assessment of severe COVID-19 phenotypes entailed 18,152 cases and 1,145,546 controls, specifically targeting individuals who either succumbed to the illness or necessitated respiratory support (13). Detailed information regarding these parameters can be found in **Tables 1**, **2**.

**Table 1:** An overview of the summary data from the GWAS used in the present study.

Trait	Ancestry	Ncase	Ncontrol	Sample size	PMID/URL
COVID-19 susceptibility	Mixed	159,840	2,782,977	2,942,817	https://www.covid19hg.org/results/r7/
COVID-19 hospitalization	Mixed	44,986	2,356,386	2,401,372	https://www.covid19hg.org/results/r7/
COVID-19 severity	Mixed	18,152	1,145,546	1,163,698	https://www.covid19hg.org/results/r7/
NMOSD	European	215	1,244	1,459	29769526
AQP4+NMOSD	European	132	1,244	1,376	29769526
AQP4-NMOSD	European	83	1,244	1,327	29769526

Mixed represents a wide range of countries from around the world, but the European population is the majority (87.2% - 99.3%). PMID/URL: the PubMed ID or web link. NMOSD, neuromyelitis optica spectrum disorder; AQP4, aquaporin-4; +/-, Positive or negative antibody.

**Table 2:** Definition of case and control for the COVID-19 phenotype.

Trait	Definition for case	Definition for control
COVID-19 susceptibility (COVID-19 infection)	a positive SARS-CoV-2 infection (e.g., RNA RT-PCR or serloogy test), electronic health record evidence of SARS-CoV-2 infection (using International Classification of Diseases or physician notes), or self-reported infections from the patients	any individuals without a history of COVID-19
COVID-19 hospitalization	hospitalized patients with COVID-19	any individuals not experiencing a hospitalization for COVID-19, which includes those without COVID-19
COVID-19 severity (severe COVID-19)	hospitalized individuals with COVID-19 who died or required respiraonry support. Respiraonry support was defined as intubation, continuous positive airway pressure (CPAP), bilevel positive airway pressure (BiPAP), continuous external negative pressure, or high-flow nasal cannula.	individuals without severe COVID-19 (including those without COVID-19)

### Outcome sources

The NMOSD trait analyzed in this study was derived from an extensive GWAS dataset, comprising 215 individuals diagnosed with NMOSD, including 132 with aquaporin-4 antibody-positive NMOSD (AQP4+NMOSD), 83 with aquaporin-4 antibody-negative NMOSD (AQP4-NMOSD), and a control group of 1,244 healthy individuals (14). The researchers conducted a preliminary study (Stage I) that included 86 individuals diagnosed with NMOSD and a control group of 460 healthy participants. In the subsequent phase (Stage II), they expanded their investigation to include 129 individuals with NMOSD and an additional 784 healthy controls. Following these phases, a comprehensive meta-analysis was conducted. Through meticulous analysis, this study successfully identified two distinct signals within the major histocompatibility complex region that are significantly associated with NMOSD. Notably, the findings revealed that AQP4+NMOSD exhibits a greater genetic resemblance to systemic lupus erythematosus (SLE) than to multiple sclerosis (MS). The clinical characteristics of the NMOSD sample at both stages are shown in **Table 3**.

**Table 3:** Clinical picture in the NMOSD patients.

	Stage I	Stage II
**Ancestry**	European	European
**Age**	45.5 years	46 years
**Female-to-male ratio**	42:1	NA
**Ncase**	total: 86; AQP4+: 66; AQP4-: 20	total: 129; AQP4+: 66; AQP4-: 63
**Ncontrol**	460	784
**Definition for case**	Patients from the University of Texas Southwestern and the Accelerated Cure Project who met the 2006 NMO diagnostic criteria.	Patients from the Accelerated Cure Project extension who meet the 2006 NMO diagnostic criteria.
**Definition for control**	normal individuals from the Genomic Psychiatry Cohort	neurological normal individuals from Coriell collections
Type of first symptoms		
**Visual**	total: 28/86; AQP4+: 21/66; AQP4-: 7/20	NA
**Spinal**	total: 46/86; AQP4+: 39/66; AQP4-: 7/20	NA
**Both**	total: 8/86; AQP4+: 4/66; AQP4-: 4/20	NA
**Smoker**	total: 57/86; AQP4+: 43/66; AQP4-: 14/20	NA
**Years Diagnosed (mean ± standard deviation)**	total: NA; AQP4+: 2.26 ± 2.19; AQP4-: 2.18 ± 2.39	NA
**Years Symptoms** **(mean ± standard deviation)**	total: NA; AQP4+: 5.79 ± 6.12; AQP4-: 6.47 ± 7.21	NA

NA, not available; AQP4, aquaporin-4; +/-, Positive or negative antibody.

### Selection of instrumental variables

First, we identified SNPs that exhibited strong associations (P < 5E-8) with COVID-19 phenotypes. Second, a clumping procedure was applied to SNPs representing the COVID-19 phenotypes, employing a cut-off value of R^2^ < 0.001 and a clumping window of 10,000 kb. Third, SNP proxies were used if no IVs were extracted from the outcome trait (minimum LD R^2^ value 0.8). Fourth, we harmonized the selected IVs and removed palindromic SNPs to avoid potential reverse causality. In addition, the F-statistic was computed for each SNP to address weak instrument bias (10). F-statistic > 10 implies strong instrument strength. The F-statistic was derived using the following formula: R^2^ × (N-k-1)/[(1-R^2^) × k], where N represents the sample size of the COVID-19 phenotype GWAS, k denotes the number of SNPs, and R^2^ signifies the proportion of variability in the COVID-19 phenotype that can be explained by each SNP. Additionally, R^2^ was computed using the formula: 2 × beta^2^ × (1-EAF) × EAF, with EAF representing the effect allele frequency and beta representing the estimated genetic effect of each SNP on the COVID-19 phenotype (15, 16).

### MR analysis and sensitivity analysis

The primary estimation of causal effects was conducted using the random-effects inverse-variance weighted (IVW) method, which combines the Wald ratio of each SNP pair outcome to generate an overall causal estimate (17). We also employed MR-Egger, weighted median, simple mode, and weighted mode methods to complement the IVW. The Cochran’s Q test of the IVW approach was used to investigate heterogeneity. To detect horizontal pleiotropy, we utilized the MR-Egger intercept test and MR Pleiotropy Residual Sum and Outlier test (MR-PRESSO). Robustness and consistency of the MR results were evaluated through leave-one-out analysis and funnel plot (18). In addition, we utilized a widely used tool (https://shiny.cnsgenomics.com/mRnd/) to evaluate the statistical power of our MR estimates. Although any violations of the MR hypothesis were evaluated in extensive sensitivity analyses, we used the PhenoScanner tool (19, 20) to assess whether genetic variants were associated with potential confounders including age, sex, and autoimmune disease (21–23). Multiple comparisons were corrected by Bonferroni measures (P=0.05/9 = 0.001). P < 0.001 was considered statistically significant. P < 0.05 was regarded as nominally significant. Our study followed the STROBE-MR Statement (24). All statistical analyses were performed using the TwoSampleMR package (v 0.5.6) within the R software (v 4.2.1).

## Results

In our MR studies, we obtained strongly correlated (P < 5E-8) independent (R^2^ < 0.001) SNPs for exposure. All F-statistics were greater than 10 (from 729 to 99007). These SNPs explained between 1.73% and 24.08% of the variance of exposure. All MR estimates of power were greater than 0.8, representing sufficient (**Supplementary Table S1**).

### MR effect of COVID-19 phenotypes on NMOSD

**Figure 2:**
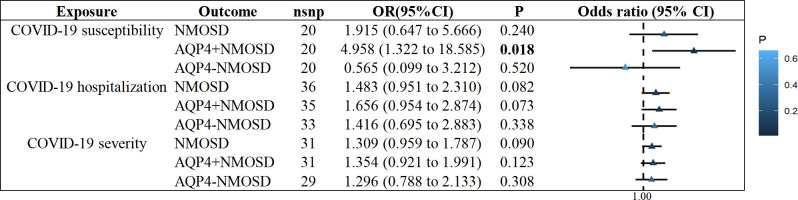
IVW results of COVID−19 phenotypes on risk of NMOSD. IVW, inverse-variance weighted method; NMOSD, neuromyelitis optica spectrum disease.

We observed no statistically significant effect of COVID-19 susceptibility on the increased risk of NMOSD or AQP4-NMOSD (NMOSD: OR = 1.915, 95% CI: 0.647 - 5.667, P = 0.240; AQP4-NMOSD: OR = 0.565, 95% CI: 0.099 to 3.212, P = 0.520; **Supplementary Figures S1-3**). Similarly, we found no evidence to support a causal association between COVID-19 hospitalization and severity on NMOSD, AQP4+NMOSD or AQP4-NMOSD (**Figure 2**). Interestingly, genetic liability to COVID-19 susceptibility was nominally associated with AQP4+NMOSD by implementing the IVW approach (OR = 4.958, 95% CI: 1.322-18.585, P = 0.018; **Figure 2**), consistent with MR Egger, weighted median and weighted mode (**Supplementary Figures S1-3**).

### Sensitivity analysis

In our comprehensive MR analyses, the Cochran’s Q test yielded p-values greater than 0.05, suggesting the absence of heterogeneity. The Egger intercept, approaching zero with a p-value exceeding 0.05, indicated no potential horizontal pleiotropy. Furthermore, the MR-PRESSO test detected no outliers of horizontal pleiotropy that could compromise our MR estimates (all Global Test p-values > 0.05) (**Table 4**). The robustness of our findings was confirmed by leave-one-out analyses, which demonstrated that MR estimates were not influenced by individual SNPs. Funnel plots were almost symmetrical with the IVW method (**Supplementary Figures S1-3**). These results further strengthen the robustness and consistency of MR results.

**Table 4:** Sensitivity analysis of the causal association between COVID-19 phenotype and the risk of NMOSD.

MR analysis		Heterogeneity		Horizontal pleiotropy		MR-PRESSO	
Exposure	Outcome	Cochran’s Q	P value	Egger intercept	P value	P value	Outlier
COVID-19 susceptibility	NMOSD	13.6	0.805	-0.0253	0.595	0.832	0
	AQP4+NMOSD	16.2	0.647	-0.0477	0.417	0.686	0
	AQP4-NMOSD	14	0.784	0.0135	0.857	0.83	0
COVID-19 hospitalization	NMOSD	31.2	0.653	0.0192	0.628	0.677	0
	AQP4+NMOSD	30.6	0.633	0.0083	0.865	0.658	0
	AQP4-NMOSD	20.7	0.938	0.0258	0.679	0.947	0
COVID-19 severity	NMOSD	19.3	0.933	0.0481	0.263	0.932	0
	AQP4+NMOSD	20.3	0.907	0.0517	0.332	0.905	0
	AQP4-NMOSD	18.9	0.9	0.0352	0.6	0.91	0

MR, Mendelian randomization; NMOSD, neuromyelitis optica spectrum disorder; AQP4, aquaporin-4 antibody; +/-, positive/negative.

### Confounding analysis

Although extensive sensitivity analyses indicated no bias in MR estimates, we manually retrieved potential confounders using the Phenoscanner online tool. We identified associations of rs505922 with Graves’ disease, rs9916158 with ulcerative colitis, rs1634761 and rs1128175 with self-reported MS, and rs13107325, rs914615 and rs646327 with Crohn’s disease. After excluding these SNPs, the causal association of COVID-19 susceptibility with AQP4+NMOSD remained nominally significant (IVW: OR = 5.090, 95% CI: 1.151-22.505, P = 0.032). The remaining MR analyses were consistent with previous results (**Supplementary Table S2**).

## Discussion

To our knowledge, we have comprehensively assessed the causal association between COVID-19 phenotypes and NMOSD for the first time. In this MR study, our findings showed that genetically predicted COVID-19 susceptibility was associated with a high risk of AQP4+ NMOSD. Extensive sensitivity analysis was consistent with the primary results, consolidating the stability and consistency of the MR analysis.

Accumulating evidence shows an inextricable association between SARS-CoV-2 infection and neuroimmune diseases (4). The underlying pathogenesis is that SARS-CoV-2 infection of multiple cells, including macrophages, neutrophils, and dendritic cells, induces an immune response in T cells and B cells by producing interferon and drives the accumulation of pro-inflammatory chemokines and cytokines, creating a “cytokine storm” that invades and damages the central and peripheral nervous system, leading to neuroinflammation and demyelination (4, 25). The development of these processes is related to the mechanisms by which viruses trigger autoimmunity including viral mimicry, propagation of epitopes, activation of bystanders, and presentation of cryptogenic antigens (26).

However, current studies on COVID-19 phenotypes in NMOSD are limited to a few case reports or case series, making it difficult to accurately assess the association between them. Several cases of optic neuritis or transverse myelitis accompanying SARS-CoV-2 infection have been reported (27–30). Other individuals have developed MRI manifestations consistent with NMOSD and positive AQP4 IgG after receiving the COVID-19 vaccine (31–33). More importantly, an increasing number of case reports demonstrate the development of NMOSD in patients after COVID-19 infection, with a proportion detecting positive AQP4 antibodies (8, 9, 34–38). Our study found the causal effect of COVID-19 susceptibility on AQP4+NMOSD, which supports previous observational findings. But the underlying mechanisms need to be clarified. Notably, although we did not find evidence for causal effects of COVID-19 phenotypes on NMOSD or AQP4-NMOSD, we had sufficient power (>0.8) to assess these associations in the current study.

To the best of our knowledge, there are currently no studies utilizing MR approach to investigate the causal effects of other infections on NMOSD. However, a recent review has highlighted the association between COVID-19 and central nervous system demyelination, emphasizing the critical importance of establishing a causal relationship (4). MS, as a distinct entity of demyelinating disorders separate from NMOSD, has received considerable attention. Despite several studies using MR methods to investigate the association between various COVID-19 phenotypes and MS, research results have yielded inconsistent conclusions. For example, Shang et al. investigated the causal effect of COVID-19 using phenotype data from the COVID-19 Host Genetics Initiative (Release 6), but found no positive association with MS (39), in agreement with the conclusions of Larsson et al. (40). However, using the largest available dataset from the COVID-19 Host Genetics Initiative (Release 7), Baranova et al. showed that genetic susceptibility to hospitalized COVID-19 had a causal effect on MS (OR: 1.15, 95%CI: 1.02-1.30, p = 0.022, FDR = 0.044) (41). The use of the most comprehensive COVID-19 phenotype data to date by Baranova et al. makes their findings more reliable and consistent with certain case reports or case series observations (6, 42, 43).

It is important to note that although MR studies have genetically confirmed the potential causal relationship between COVID phenotypes and NMOSD or MS, further validation of these findings is needed, particularly in the absence of large cohort studies. This means that MR studies can be considered as preliminary research that to some extent fills these gaps.

The advantage of the current study is that compared to traditional observational studies, we have utilized MR methods to minimize the interference of reverse causality and residual confounders. Sensitivity analysis also supported the robustness of MR estimates.

Nevertheless, several limitations exist in our study. First, our GWAS data were mainly from European populations, so estimates of the overall population still need to be clarified. Second, we could not specify the proportion of participants with a possible overlap in exposure and outcome. Third, due to the different origins of data on different COVID-19 phenotypes, individuals affected by COVID-19 may manifest phenotypic variation influenced by local factors, such as the standard of health care. Fourth, the genetic explanatory power of significant SNPs is not entirely satisfactory, suggesting the potential involvement of other factors in the occurrence of NMOSD. Due to the large sample size, this study achieved an ideal statistical power (>0.8), which implies the reliability of the statistical conclusions derived from the present research. Fifth, due to data limitations, it becomes challenging to present the clinical characteristics of COVID-19 phenotype data, such as age and gender. This limitation may hinder the clinical applicability. Ongoing MR studies have begun preliminary explorations of the causal effects of different COVID-19 phenotypes on NMOSD, which warrant further verification through cohort studies.

In conclusion, we provide strong evidence supporting the genetic liability to COVID-19 susceptibility was positively associated with the high risk of AQP4+ NMOSD. These contribute to our understanding of the prevention, diagnosis, treatment, and potential pathogenesis of NMOSD during the COVID-19 pandemic. Further studies are still warranted.

## Data availability statement

The original contributions presented in the study are included in the article/**Supplementary Material**. Further inquiries can be directed to the corresponding author.

## Author contributions

The overall research was conceptualized and designed by HZ. DS performed the data analysis and contributed to the writing and revision of the manuscript. All authors participated in data collection and monitoring. The submitted version of the manuscript was reviewed and approved by all authors.

## Supplementary material

Supplementary Table S1 Variance and power of COVID-19 phenotypes and neuromyelitis optica spectrum disorder.

Supplementary Table S2 MR reanalysis after removal of confounding factors.

Supplementary Figure S1 MR estimation and sensitivity analysis of COVID-19 phenotypes on neuromyelitis optica spectrum disorder (NMOSD).

Supplementary Figure S2 MR estimation and sensitivity analysis of COVID-19 phenotypes on aquaporin-4 antibody-positive NMOSD (AQP4+NMOSD).

Supplementary Figure S3 MR estimation and sensitivity analysis of COVID-19 phenotypes on aquaporin-4 antibody-negative NMOSD (AQP4-NMOSD).
