# Supplementary material for: COVID-19 and the risk of neuromyelitis optica spectrum disorder: a Mendelian randomization study
Source: Front Immunol. 2023 Jul 27;14:1207514. doi: 10.3389/fimmu.2023.1207514 (PMC10414539; doi:10.3389/fimmu.2023.1207514)
Supplement: Supplementary Table S1 — Variance and power of COVID-19 phenotypes and neuromyelitis optica spectrum disorder. [file DataSheet_1.docx]

**SUPPLEMENTAL MATERIAL**

**COVID-19 and the risk of neuromyelitis optica spectrum disorder: a Mendelian randomization study**

Dongren Sun^1^, Qin Du^1^, Rui Wang^1^, Ziyan Shi^1^, Hongxi Chen^1^, Hongyu Zhou^1,*^

^1^Department of Neurology, West China Hospital, Sichuan University, Guo Xuexiang #37, Chengdu 610041, China.

*Corresponding author:

Name: Hongyu Zhou

E-mail address: zhouhy@scu.edu.cn

**Contents**

**Supplementary Table S1.** Variance and power of COVID-19 phenotypes and neuromyelitis optica spectrum disorder.

**Supplementary Table S2.** MR reanalysis after removal of confounding factors.

**Supplementary Figure S1.** MR estimation and sensitivity analysis of COVID-19 phenotypes on neuromyelitis optica spectrum disorder (NMOSD).

**Supplementary Figure S2.** MR estimation and sensitivity analysis of COVID-19 phenotypes on aquaporin-4 antibody-positive NMOSD (AQP4+NMOSD).

**Supplementary Figure S3.** MR estimation and sensitivity analysis of COVID-19 phenotypes on aquaporin-4 antibody-negative NMOSD (AQP4-NMOSD).

**Supplementary Table S1.** Variance and power of COVID-19 phenotypes and neuromyelitis optica spectrum disorder.

| **Exposure** | **Outcome** | **Variance** | **Power** |
| --- | --- | --- | --- |
| COVID-19 susceptibility | NMOSD | 0.0174 | 1.00 |
|  | AQP4+NMOSD | 0.0174 | 1.00 |
|  | AQP4-NMOSD | 0.0174 | 1.00 |
| COVID-19 hospitalization | NMOSD | 0.1155 | 1.00 |
|  | AQP4+NMOSD | 0.1142 | 1.00 |
|  | AQP4-NMOSD | 0.1100 | 1.00 |
| COVID-19 severity | NMOSD | 0.2408 | 1.00 |
|  | AQP4+NMOSD | 0.2408 | 1.00 |
|  | AQP4-NMOSD | 0.2249 | 1.00 |

**Supplementary Table S2 .** MR reanalysis after removal of confounding factors.

| **Exposure** | **Outcome** | **Method** | **P** | **OR** | **95%CI** | |
| --- | --- | --- | --- | --- | --- | --- |
| COVID-19 susceptibility | NMOSD | MR Egger | 0.353 | 2.580 | 0.369 | 18.025 |
|  |  | Weighted median | 0.159 | 3.220 | 0.634 | 16.354 |
|  |  | IVW | 0.399 | 1.677 | 0.505 | 5.574 |
|  |  | Simple mode | 0.761 | 1.559 | 0.093 | 26.124 |
|  |  | Weighted mode | 0.273 | 2.252 | 0.553 | 9.168 |
|  | AQP4+NMOSD | MR Egger | 0.109 | 7.702 | 0.746 | 79.550 |
|  |  | Weighted median | 0.026 | 9.077 | 1.309 | 62.954 |
|  |  | IVW | **0.032** | 5.090 | 1.151 | 22.505 |
|  |  | Simple mode | 0.949 | 0.905 | 0.045 | 18.064 |
|  |  | Weighted mode | 0.060 | 7.967 | 1.076 | 59.000 |
|  | AQP4-NMOSD | MR Egger | 0.631 | 0.440 | 0.017 | 11.664 |
|  |  | Weighted median | 0.772 | 0.678 | 0.049 | 9.392 |
|  |  | IVW | 0.615 | 0.601 | 0.083 | 4.374 |
|  |  | Simple mode | 0.785 | 1.678 | 0.043 | 64.891 |
|  |  | Weighted mode | 0.727 | 0.624 | 0.046 | 8.448 |
| COVID-19 hospitalization | NMOSD | MR Egger | 0.467 | 1.344 | 0.611 | 2.958 |
|  |  | Weighted median | 0.688 | 1.149 | 0.583 | 2.265 |
|  |  | IVW | 0.135 | 1.410 | 0.899 | 2.211 |
|  |  | Simple mode | 0.496 | 1.525 | 0.459 | 5.069 |
|  |  | Weighted mode | 0.628 | 1.201 | 0.577 | 2.497 |
|  | AQP4+NMOSD | MR Egger | 0.261 | 1.766 | 0.667 | 4.677 |
|  |  | Weighted median | 0.425 | 1.403 | 0.611 | 3.222 |
|  |  | IVW | 0.157 | 1.497 | 0.856 | 2.617 |
|  |  | Simple mode | 0.511 | 1.671 | 0.368 | 7.583 |
|  |  | Weighted mode | 0.323 | 1.526 | 0.669 | 3.483 |
|  | AQP4-NMOSD | MR Egger | 0.921 | 1.068 | 0.293 | 3.893 |
|  |  | Weighted median | 0.933 | 1.047 | 0.354 | 3.094 |
|  |  | IVW | 0.294 | 1.473 | 0.715 | 3.035 |
|  |  | Simple mode | 0.824 | 1.248 | 0.181 | 8.625 |
|  |  | Weighted mode | 0.987 | 0.991 | 0.360 | 2.733 |
| COVID-19 severity | NMOSD | MR Egger | 0.980 | 1.007 | 0.583 | 1.740 |
|  |  | Weighted median | 0.748 | 1.080 | 0.675 | 1.729 |
|  |  | IVW | 0.090 | 1.309 | 0.959 | 1.787 |
|  |  | Simple mode | 0.180 | 1.909 | 0.758 | 4.807 |
|  |  | Weighted mode | 0.605 | 1.134 | 0.708 | 1.817 |
|  | AQP4+NMOSD | MR Egger | 0.944 | 1.025 | 0.522 | 2.013 |
|  |  | Weighted median | 0.372 | 1.302 | 0.729 | 2.325 |
|  |  | IVW | 0.123 | 1.354 | 0.921 | 1.991 |
|  |  | Simple mode | 0.238 | 2.001 | 0.647 | 6.187 |
|  |  | Weighted mode | 0.374 | 1.340 | 0.709 | 2.531 |
|  | AQP4-NMOSD | MR Egger | 0.903 | 1.058 | 0.433 | 2.586 |
|  |  | Weighted median | 0.877 | 1.059 | 0.513 | 2.184 |
|  |  | IVW | 0.271 | 1.327 | 0.802 | 2.197 |
|  |  | Simple mode | 0.347 | 1.888 | 0.514 | 6.932 |
|  |  | Weighted mode | 0.816 | 1.092 | 0.525 | 2.270 |

**Supplementary Figure S1.** MR estimation and sensitivity analysis of COVID-19 phenotypes on neuromyelitis optica spectrum disorder (NMOSD).


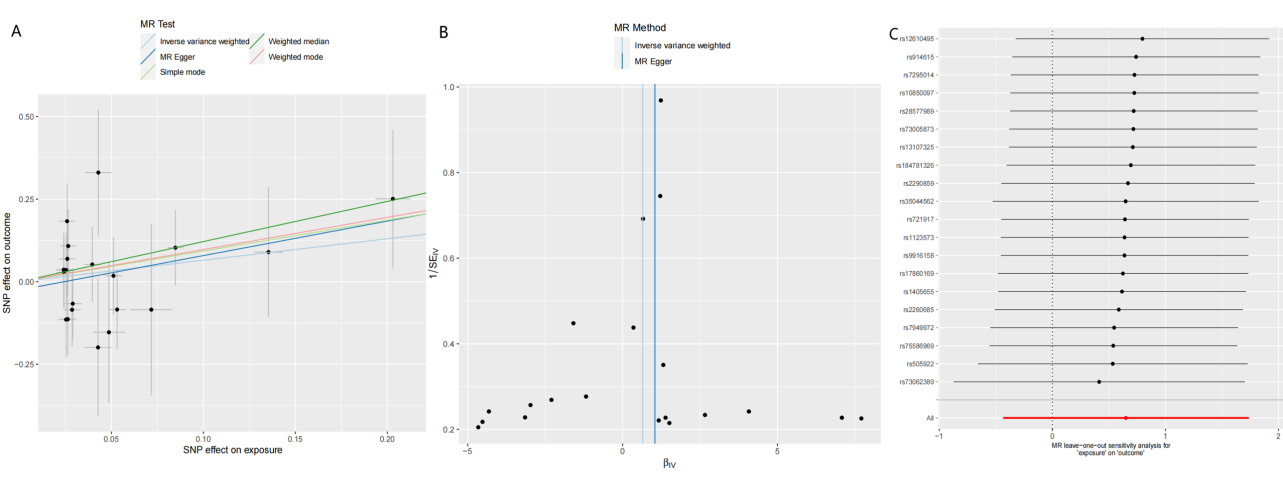


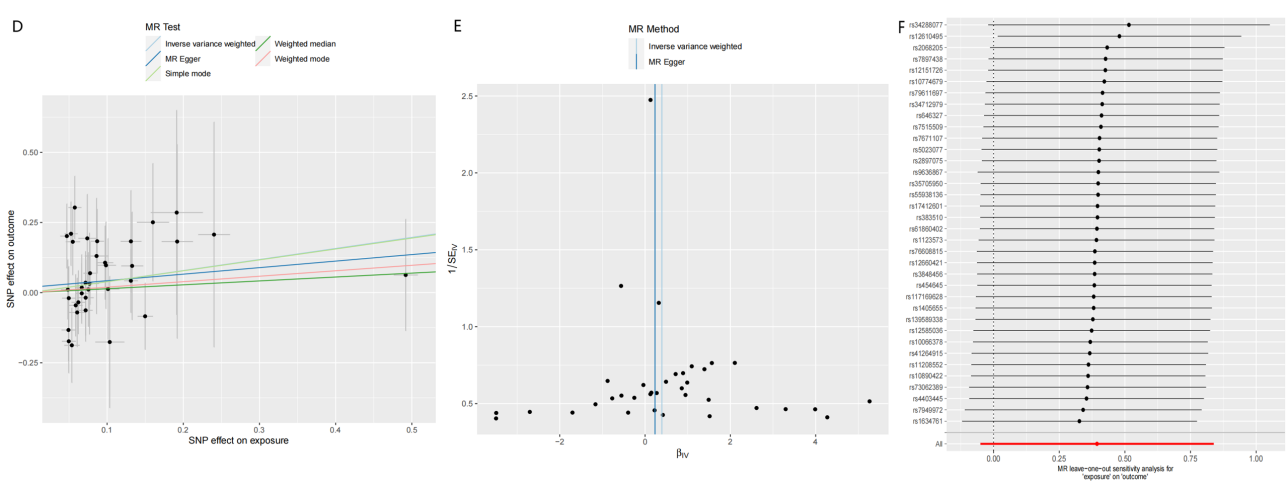


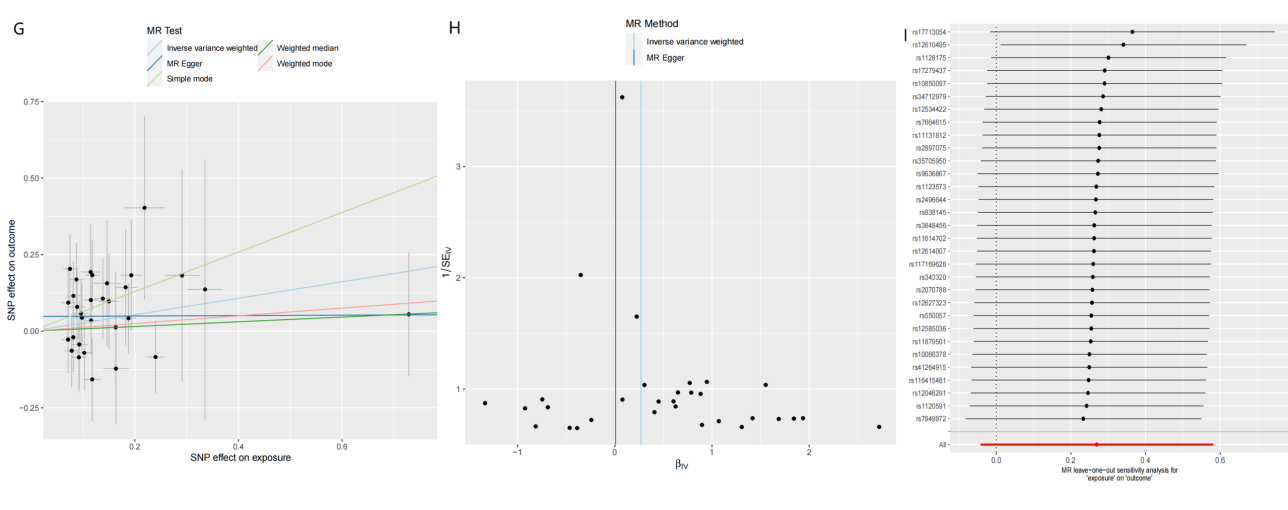


1. Scatter plots from genetically predicted COVID-19 susceptibility on NMOSD; (B) Funnel plot from genetically predicted COVID-19 susceptibility on NMOSD; (C) Leave-one-out plot from genetically predicted COVID-19 susceptibility on NMOSD; (D) Scatter plots from genetically predicted COVID-19 hospitalization on NMOSD; (E) Funnel plot from genetically predicted COVID-19 hospitalization on NMOSD; (F) Leave-one-out plot from genetically predicted COVID-19 hospitalization on NMOSD; (G) Scatter plots from genetically predicted COVID-19 severity on NMOSD; (H) Funnel plot from genetically predicted COVID-19 severity on NMOSD; (I) Leave-one-out plot from genetically predicted COVID-19 severity on NMOSD.

**Supplementary Figure S2.** MR estimation and sensitivity analysis of COVID-19 phenotypes on aquaporin-4 antibody-positive NMOSD (AQP4+NMOSD) .


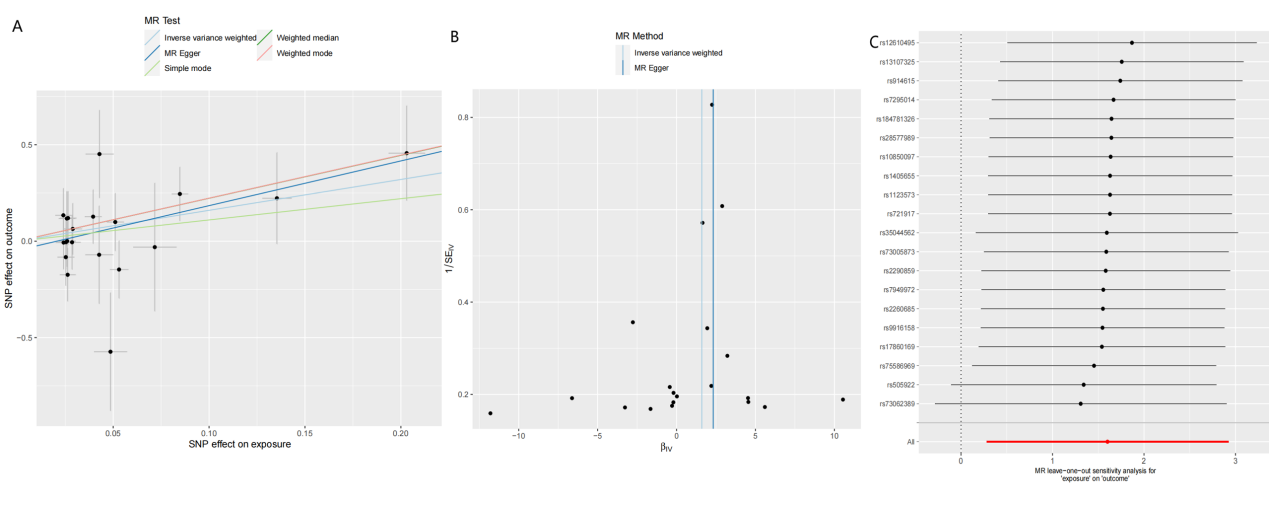


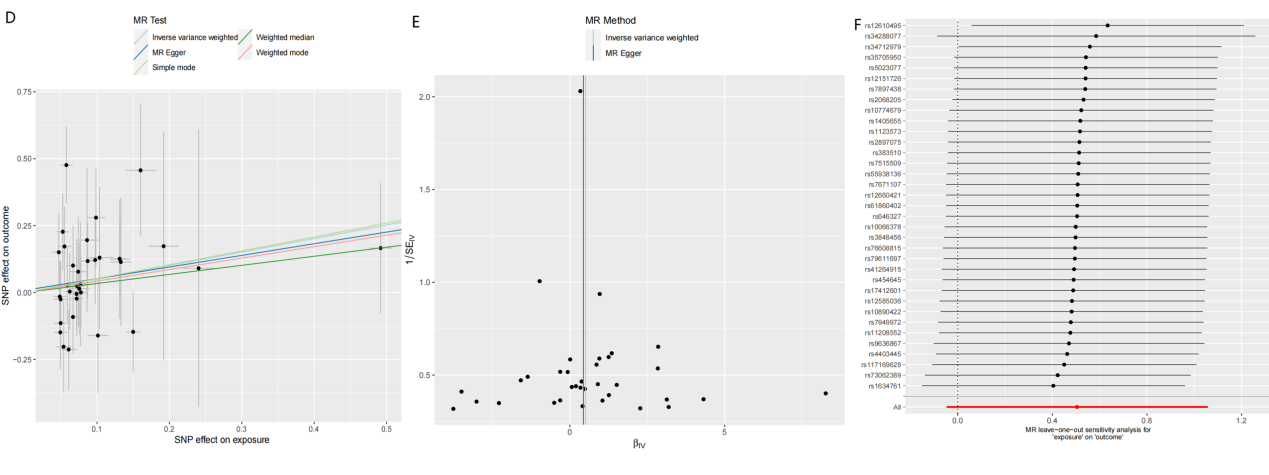


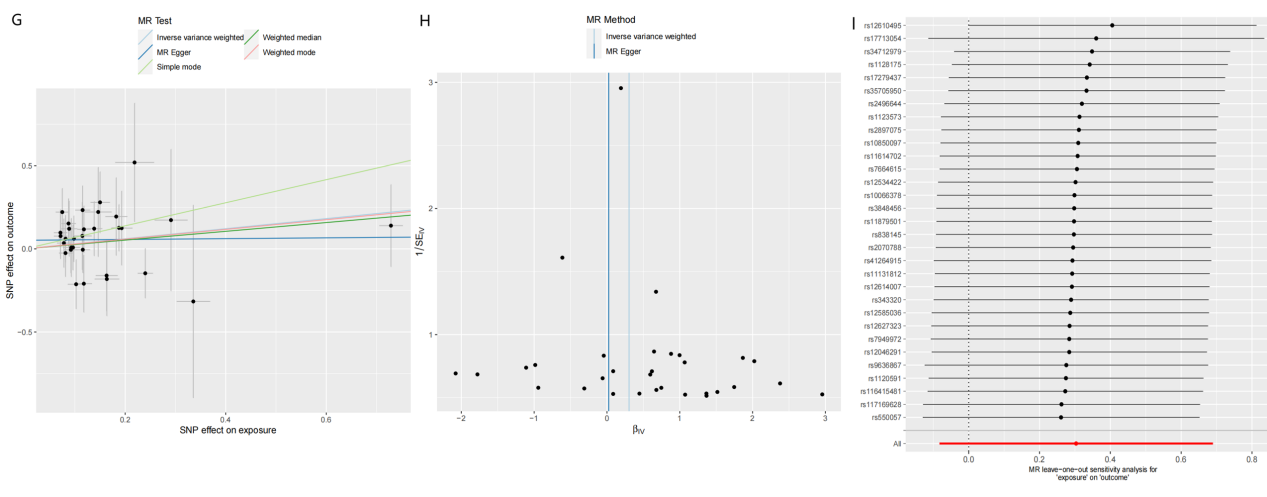


1. Scatter plots from genetically predicted COVID-19 susceptibility on AQP4+NMOSD; (B) Funnel plot from genetically predicted COVID-19 susceptibility on AQP4+NMOSD; (C) Leave-one-out plot from genetically predicted COVID-19 susceptibility on AQP4+NMOSD; (D) Scatter plots from genetically predicted COVID-19 hospitalization on AQP4+NMOSD; (E) Funnel plot from genetically predicted COVID-19 hospitalization on AQP4+NMOSD; (F) Leave-one-out plot from genetically predicted COVID-19 hospitalization on AQP4+NMOSD; (G) Scatter plots from genetically predicted COVID-19 severity on AQP4+NMOSD; (H) Funnel plot from genetically predicted COVID-19 severity on AQP4+NMOSD; (I) Leave-one-out plot from genetically predicted COVID-19 severity on AQP4+NMOSD.

**Supplementary Figure S3.** MR estimation and sensitivity analysis of COVID-19 phenotypes on aquaporin-4 antibody-negative NMOSD (AQP4-NMOSD).


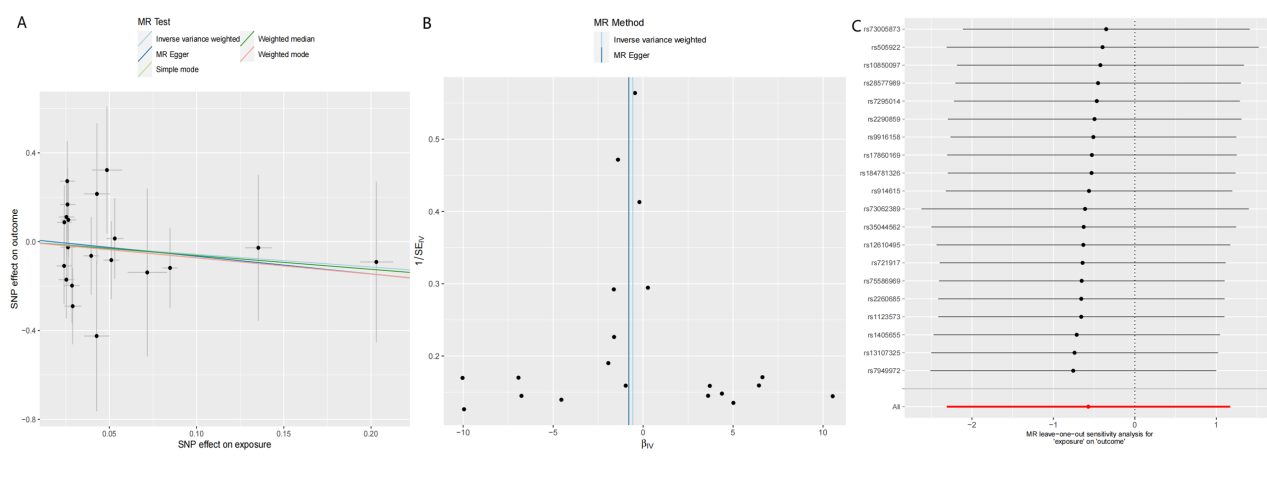

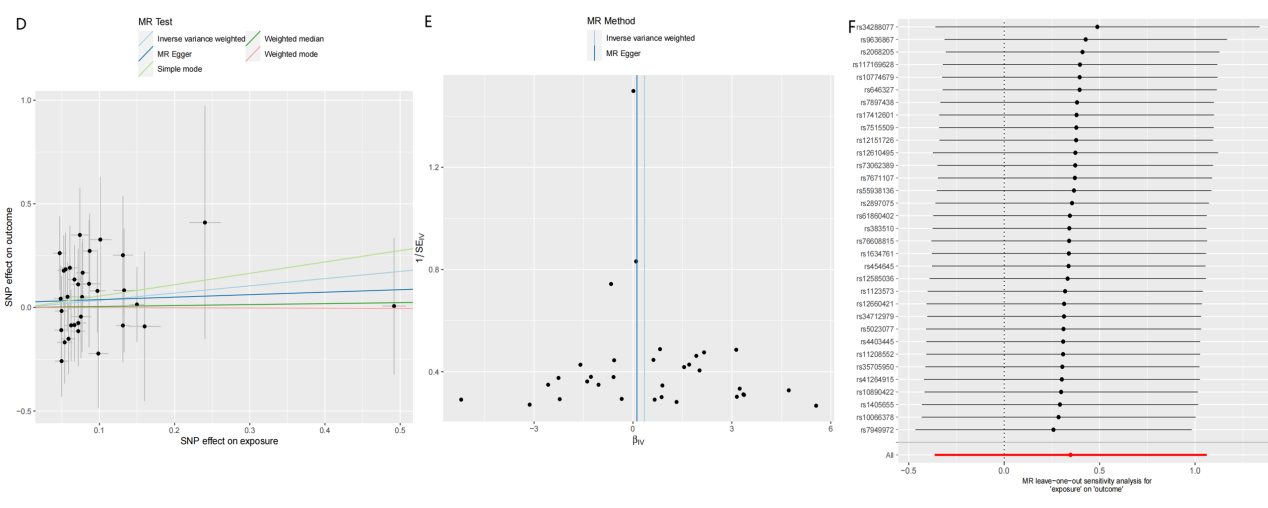


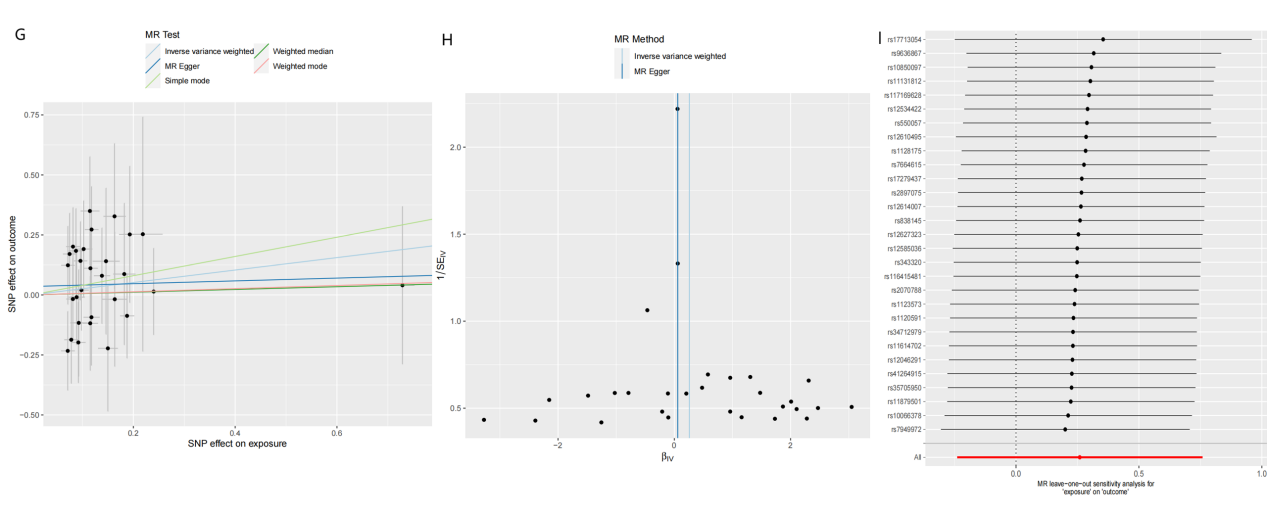


(A) Scatter plots from genetically predicted COVID-19 susceptibility on AQP4-NMOSD; (B) Funnel plot from genetically predicted COVID-19 susceptibility on AQP4-NMOSD; (C) Leave-one-out plot from genetically predicted COVID-19 susceptibility on AQP4-NMOSD; (D) Scatter plots from genetically predicted COVID-19 hospitalization on AQP4-NMOSD; (E) Funnel plot from genetically predicted COVID-19 hospitalization on AQP4-NMOSD; (F) Leave-one-out plot from genetically predicted COVID-19 hospitalization on AQP4-NMOSD; (G) Scatter plots from genetically predicted COVID-19 severity on AQP4-NMOSD; (H) Funnel plot from genetically predicted COVID-19 severity on AQP4-NMOSD; (I) Leave-one-out plot from genetically predicted COVID-19 severity on AQP4-NMOSD.
